# Graft protective effects and donor-specific antibody suppression by CD4^+^CD25^+^Foxp3^+^ regulatory T cell induced by HMG-CoA reductase inhibitor rosuvastatin in a murine heart transplant model

**DOI:** 10.1186/s13019-024-02888-4

**Published:** 2024-06-25

**Authors:** Kazuhito Iguchi, Yasuto Yamamoto, Masateru Uchiyama, Hisanori Masaoka, Masahiro Nakamura, Hiroyuki Shizuka, Tomohiro Imazuru, Tomoki Shimokawa

**Affiliations:** Department of Cardiovascular Surgery, Teikyo University, 2-11-1 Kaga, Itabashi-ku, Tokyo, 173-8605 Japan

**Keywords:** Rosuvastatin, HMG-CoA reductase inhibitor, Regulatory T cell, Cardiac transplant model, Mouse

## Abstract

**Background:**

We previously demonstrated that the hydroxymethylglutaryl-CoA (HMG-CoA) reductase inhibitor (statins) play an important role in the regulation of alloimmune responses. However, little is known regarding the effects of statin on allograft protection or donor-specific antibodies (DSA). In this study, we investigated the graft-protective and immunomodulatory effects of rosuvastatin in a model of fully major histocompatibility complex-mismatched murine cardiac allograft transplantation.

**Methods:**

CBA mice underwent transplantation of C57BL/6 (B6) hearts and received 50 and 500 μg/kg/day of rosuvastatin from the day of transplantation until seven days after the completion of transplantation. To confirm the requirement for regulatory T cells (Tregs), we administered an anti-interleukin-2 receptor alpha antibody (PC-61) to rosuvastatin-treated CBA recipients. Additionally, histological and fluorescent staining, cell proliferation analysis, flow cytometry, and DSA measurements were performed.

**Results:**

CBA recipients with no treatment rejected B6 cardiac graft acutely (median survival time [MST], 7 days). CBA mice treated with 500 μg/kg/day of rosuvastatin prolonged allograft survival (MSTs, 77 days). Fluorescent staining studies showed that rosuvastatin-treated recipients had strong aggregation of CD4^+^Foxp3^+^ cells in the myocardium and around the coronary arteries of cardiac allografts two weeks after grafting. Flow cytometry studies performed two weeks after transplantation showed an increased number of splenic CD4^+^CD25^+^Foxp3^+^ T cells in rosuvastatin-treated recipients. The addition of rosuvastatin to mixed leukocyte cultures suppressed cell proliferation by increasing the number of CD4^+^CD25^+^Foxp3^+^ Tregs. Additionally, Tregs suppressed DSA production in rosuvastatin-treated recipients.

**Conclusion:**

Rosuvastatin treatment may be a complementary graft-protective strategy for suppressing DSA production in the acute phase, driven by the promotion of splenic and graft-infiltrating CD4^+^CD25^+^Foxp3^+^ Tregs.

## Introduction

Organ transplantation remains the treatment of choice for patients with total loss of function in a life-sustaining organ. The use of various immunosuppressive drugs, including calcineurin inhibitors, contributes to improved clinical outcomes by suppressing T-cell-mediated graft rejection [1]. However, the complete rejection management remains to be elucidated. Even if T cell-mediated rejection can be controlled, the risk of acute rejection during the first year after heart transplantation is particularly high [2], and chronic rejection is reported to occur in approximately 3–5% of patients with transplants every year from the first year after transplantation [3]. In addition to T cell-mediated rejection, the management of antibody-mediated rejection (AMR) [4] and related coronary microvasculopathy [3] are considered important issues for graft protection. Thus, there remains a significant need for more effective therapeutic strategies to attenuate antibody-mediated graft injury and coronary vasculopathy and improve graft survival rates. In particular, as a therapeutic strategy, hydroxymethylglutaryl-CoA (HMG-CoA) reductase inhibitors (statins), which are lipid metabolism treatment drugs, are still garnering interest. The use of statins is recommended by the guidelines for the care of heart and kidney transplant recipients [5, 6] because of the significant reduction in cardiovascular events in patients after organ transplantation.

It is well known that the use of statin therapy is effective in reducing the risk of cardiovascular diseases (CVD) [7, 8]. In particular, statin-induced reduction in low-density lipoprotein cholesterol correlates with a reduction in CVD risk [9, 10]. In addition to the modulation of cholesterol levels, some reports on statin therapy have shown the regulation of natural killer cells [11] and an increase in regulatory T cells [12]. However, little is known about the role of statin therapy in protecting allograft vessels and immune cells. Clinical studies have demonstrated that the administration of pravastatin and simvastatin after heart transplantation can reduce the incidence of cardiac allograft vasculopathy and improve patient survival [13–15]. In a study of stem cell transplant recipients, rosuvastatin protected implanted mesenchymal stem cells against the post-infarct microenvironment and improved the efficacy of stem cell transplantation [16]. We previously demonstrated that statin treatment can induce graft-protective effects using morphometric analysis of neointimal formation in cardiac allografts [17]. However, we did not closely examine the immunomodulatory features of statin in our previous studies. The findings indicate that rosuvastatin can delay the progression of cardiac allograft vasculopathy through immunosuppressive effects and that rosuvastatin can be used as a mechanism-based drug. In this study, we investigated the graft-protective and immunomodulatory effects of rosuvastatin in a model of fully major histocompatibility complex (MHC)-mismatched murine cardiac allograft transplantation.

## Materials and methods

### Animals

Male CBA (H2^k^), C57BL/6 (H2^b^, [B6]), and BALB/c (H2^d^) mice aged 8 to 12 weeks were purchased from Sankyo Ltd (Tokyo, Japan), housed in conventional facilities at the Biomedical Services Unit of Teikyo University, and maintained under a 12 h–12 h light-dark cycle with ad libitum access to food and water. The experiments were performed in accordance with the guidelines for animal experimentation approved by the Animal Use and Care Committee of the university and the “Principles of Laboratory Animal Care” [18].

### Heart transplantation

Heart transplantation was performed as described previously [19]. Postoperatively, cardiac graft function was assessed daily by palpating the heart for evidence of cardiac contraction. Rejection was defined as complete cessation of the heartbeat and was confirmed by direct visualization and histological examination of the graft.

### Administration of rosuvastatin, mevalonic acid (MVA) and anti-interleukin (IL)-2 receptor alpha antibody (PC-61)

**Fig. 1:**
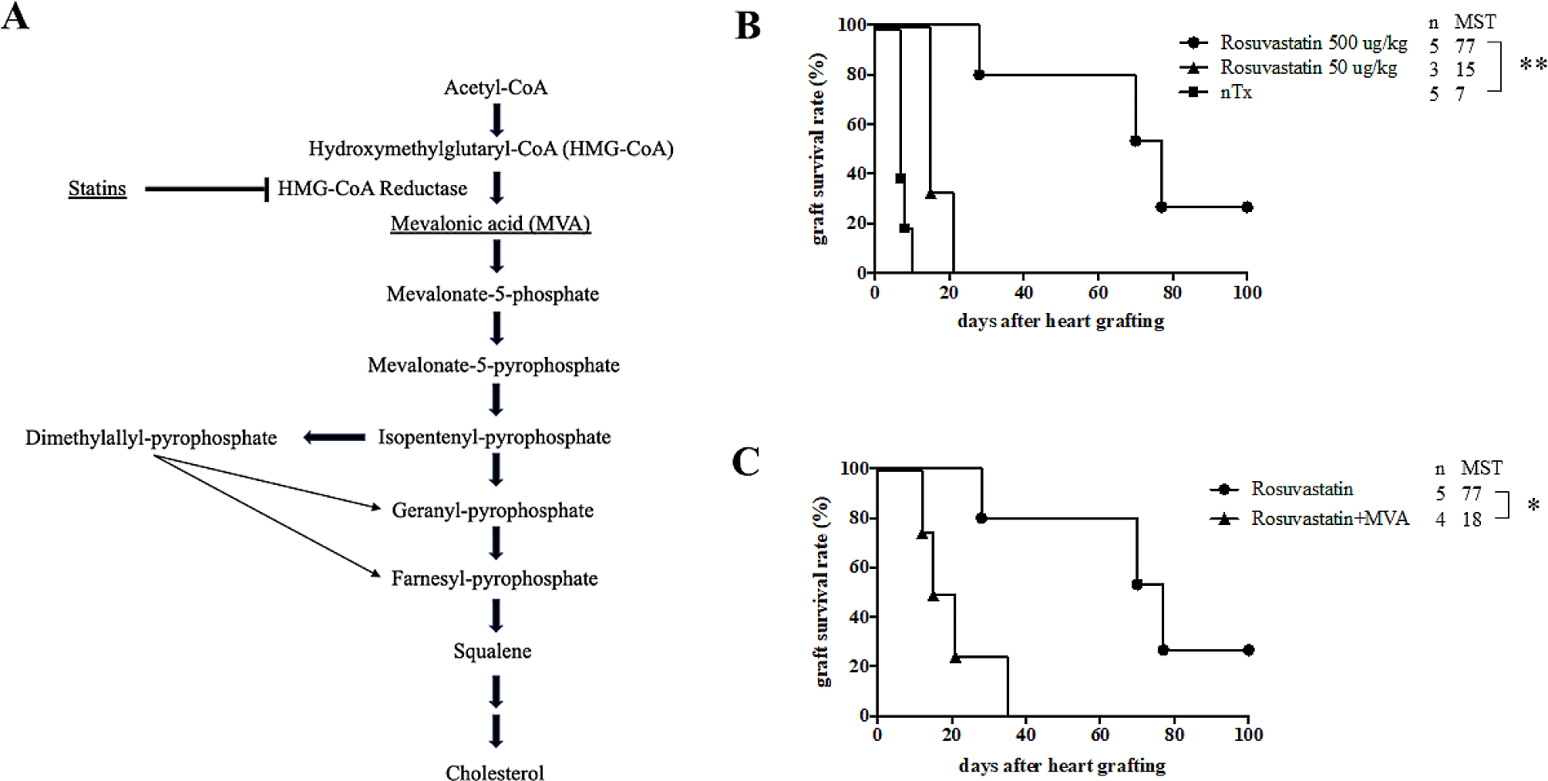
Mevalonate pathway and graft survival of CBA recipients. (**A**) The mevalonate pathway. HMG-CoA, Hydroxymethylglutaryl-CoA. MVA, mevalonic acid. (**B**) CBA recipients of a B6 heart were administered 50 and 500 μg/kg of rosuvastatin from the day of transplantation until seven days after the completion of transplantation. (**C**) CBA recipients of the B6 heart were administered 500 μg/kg of rosuvastatin and mevalonic acid (MVA) from the day of transplantation to seven days after the completion of transplantation. MST, median survival time. MVA, mevalonic acid. nTx, no-treatment. **P < 0.01 and *P < 0.05

Rosuvastatin was dissolved in distilled water and administered from the day of transplantation until seven days after transplantation. CBA recipients of a B6 heart were administered 1 mL distilled water per day (no treatment group) or 50 or 500 μg/kg/day of rosuvastatin (rosuvastatin group). To reverse the effects of rosuvastatin [20], 500 μg/kg/day of MVA (one of the intermediates in cholesterol biosynthesis pathway shown in Fig. 1A) was dissolved in normal saline and administered to rosuvastatin-treated CBA recipients from the day of transplantation until seven days after transplantation. Rosuvastatin and MVA were given orally with use of a metal tube (Thomas Scientific, Swedesboro, NJ, United States). Moreover, to confirm the requirement for regulatory T cells (Tregs) to prolong allograft survival and suppress donor-specific antibodies (DSA), anti-IL-2 receptor alpha antibody (PC-61; Abcam, Cambridge, UK) was administered to rosuvastatin-treated CBA recipients. CBA recipients treated with rosuvastatin for seven days after transplantation were intraperitoneally injected with 100 μg/d of PC-61 on days 0, 3, 6, and 9.

### Flow cytometry analysis

Splenocytes were obtained from no-treatment and rosuvastatin-treated recipients two weeks after transplantation. Additionally, splenocytes co-cultured with B6 splenocytes for seven days in mixed leukocyte culture (MLC) were obtained. These cells were stained with PE-Cy7-labeled anti-mouse CD3 (145-2C11; BD Biosciences, Franklin Lakes, NJ), FITC-labeled anti-CD4 (RM4-5; BD Biosciences), APC-Cy7-labeled anti-CD8 (53 − 6.7; Thermo Fisher Scientific, Waltham, MA), APC-labeled anti-CD25 (PC-61; BD Biosciences), and PE-labeled anti-mouse Foxp3 (FJK-16s; Thermo Fisher Scientific) monoclonal antibodies (mAb), as well as their isotype controls (Thermo Fisher Scientific). The stained cells were analyzed using a FACS CantoII system (BD Biosciences) to determine the percentage of CD4^+^ and CD8^+^ cells in CD3^+^ cells and CD25^+^Foxp3^+^ cells in CD4^+^ cells [21].

Additionally, to analyze intracellular expression in T cells, splenocytes from no-treatment and rosuvastatin-treated CBA recipients were stained with APC-labeled anti-T-bet (4B10; BioLegend, San Diego, CA) and PerCP-eFluorR-labeled anti-GATA3 (TWAJ; Thermo Fisher Scientific) mAb two weeks after transplantation.

### Measurement of DSA production

DSAs in the serum of no-treatment, rosuvastatin-treated CBA recipients, and rosuvastatin/PC-61-treated CBA recipients two weeks after transplantation were measured by flow cytometry. The complements in the sera were inactivated at 56 ℃ for 30 min. Naïve B6 or BALB/c thymocytes (2 × 10^6^/0.1 mL) were incubated with the recipient sera (10 μL) for 45 min on ice. The stained cells were washed thrice and incubated for an additional 45 min with FITC-conjugated anti-mouse IgG polyclonal antibody (Thermo Fisher Scientific). All samples were examined at the same time [21].

### Histological and fluorescent staining studies of harvest cardiac grafts

Cardiac allografts transplanted into no-treatment and rosuvastatin-treated CBA recipients were removed two weeks after grafting and studied histologically. Frozen sections (4-μm thick) were cut, mounted on silane-coated slides, and stained with hematoxylin-eosin (HE). HE staining was assessed by grading with a semi-quantitative scale for the amount of mononuclear cell infiltration (0, no infiltration; 1, faint and limited infiltration; 2, moderate infiltration; 3, severe infiltration) [22, 23]. All cardiac graft slides were blindly assessed by three trained transplant pathologists.

Fluorescent staining was performed to determine whether myocardial function in the transplanted cardiac allografts was preserved and whether CD4^+^Foxp3^+^ regulatory T cells were generated. Triple fluorescent staining of cardiac allografts was performed two weeks after grafting in rosuvastatin-treated CBA mice. Fresh 4-μm-thick graft cryosections were incubated with PE-labeled anti-mouse Foxp3 (FJK-16s; Thermo Fisher Scientific) and Alexa Fluor® 647-conjugated anti-CD4 mAb (RM4-5; BD Biosciences). Subsequently, the cryosections were incubated with rabbit anti-mouse type IV collagen polyclonal antibody (LB1403; Cosmo Bio, Tokyo, Japan), followed by incubating with AMCA-conjugated anti-rabbit Ig (711-155-152; Jackson ImmunoResearch Laboratories, West Grove, PA).

### MLC

In MLC studies, the responder cells were splenocytes from no-treatment CBA mice that had undergone transplantation of a B6 heart 14 days earlier. The stimulator cells were B6 (allogeneic) splenocytes treated with 100 μg/ml mitomycin C (Kyowa Hakko, Osaka) for 30 min at 37 ℃. Rosuvastatin (1 μmol/well) was added to MLC to assess the direct effects of this agent on cellular proliferation. The responder cells (2.5 × 10^5^/well) were co-cultured with the stimulator cells (5.0 × 10^5^/well) in complete medium in a humidified 5% CO_2_ atmosphere (CH-16 M; Hitachi, Tokyo) at 37 ℃ in 96-well, round-bottomed tissue-culture plates (Iwaki Scitech Division, Tokyo) for seven days. Proliferation was assessed using an enzyme-linked immunosorbent assay for bromodeoxyuridine incorporation (BioTrak, version 2; Amersham, Little Chalfont) according to the manufacturer’s instructions [24]. In some cases, various amounts of splenic CD4^+^CD25^+^ Treg from rosuvastatin-treated CBA recipients (0 [0], 2.5 × 10^3^/well [1:100], 5.0 × 10^3^/well [1:50], 12.5 × 10^3^/well [1:20], or 2.5 × 10^4^/well [1:10]) were added to the MLC to assess the direct effects of rosuvastatin-induced Treg purified by FACS Aria-IIIu (BD Biosciences).

### Statistical analysis

Cardiac allograft survival times in the two experimental groups were compared using the log-rank test. In the histological, cell proliferation, and flow cytometry studies, the difference between the two groups was assessed using the unpaired Student’s t-test or analysis of variance with Bonferroni correction. Statistical significance was set at *P* value < 0.05.

## Results

### Significant prolonged survival of cardiac allografts in CBA recipients treated with rosuvastatin

No treatment CBA recipients acutely rejected B6 cardiac allografts (median survival time [MST], 7 days). CBA recipients treated with 500 μg/kg/day of rosuvastatin for 7 days had significantly prolonged allograft survival (MST, 77 days; Fig. 1B). However, treatment with 50 μg/kg/day of rosuvastatin did not increase graft survival (MST, 15 days; Fig. 1B). Administration of 500 μg/kg/day of MVA for 7 days prevented CBA recipients treated with 500 μg/kg/day of rosuvastatin from prolonging allograft survival (MST, 18 days; Fig. 1C).

### Histological features of cardiac allografts in CBA recipients treated with rosuvastatin

**Fig. 2:**
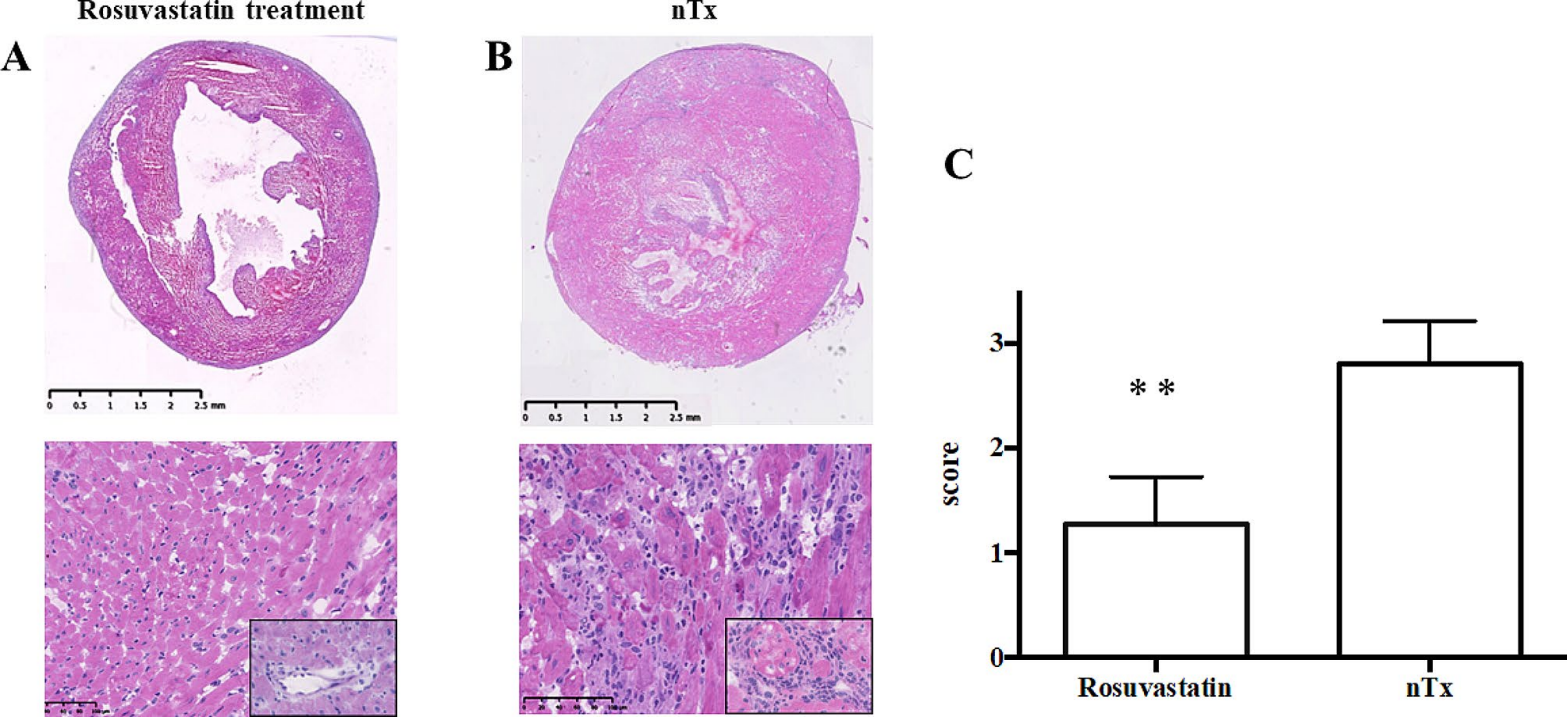
Histological studies of transplanted cardiac allografts. (**A**, **B**) Results of hematoxylin-eosin staining of representative heart cross-sections, myocardium, and coronary arteries in cardiac allografts obtained two weeks after transplantation from rosuvastatin-treated (**A**) or no-treatment group (nTx) (**B**). (**C**) Each section was assessed by grading with a semiquantitative scale for mononuclear cell infiltration by manually. nTx, no-treatment. **P < 0.01 compared with the no-treatment group

Histological examination of cardiac allografts obtained two weeks after grafting showed preserved myocardial and vessel structure in rosuvastatin-treated recipients, whereas cardiac allografts from no-treatment recipients showed myocyte damage, edema, and more aggressive inflammatory infiltrate in the process of acute rejection (Fig. 2A-B). Moreover, there was a significant difference in the graft score grade on a semi-quantitative scale between no-treatment and rosuvastatin-treated allografts (Fig. 2C).

### Prominent accumulation of CD4^+^Foxp3^+^ cells in cardiac allografts from rosuvastatin-treated mice

**Fig. 3:**
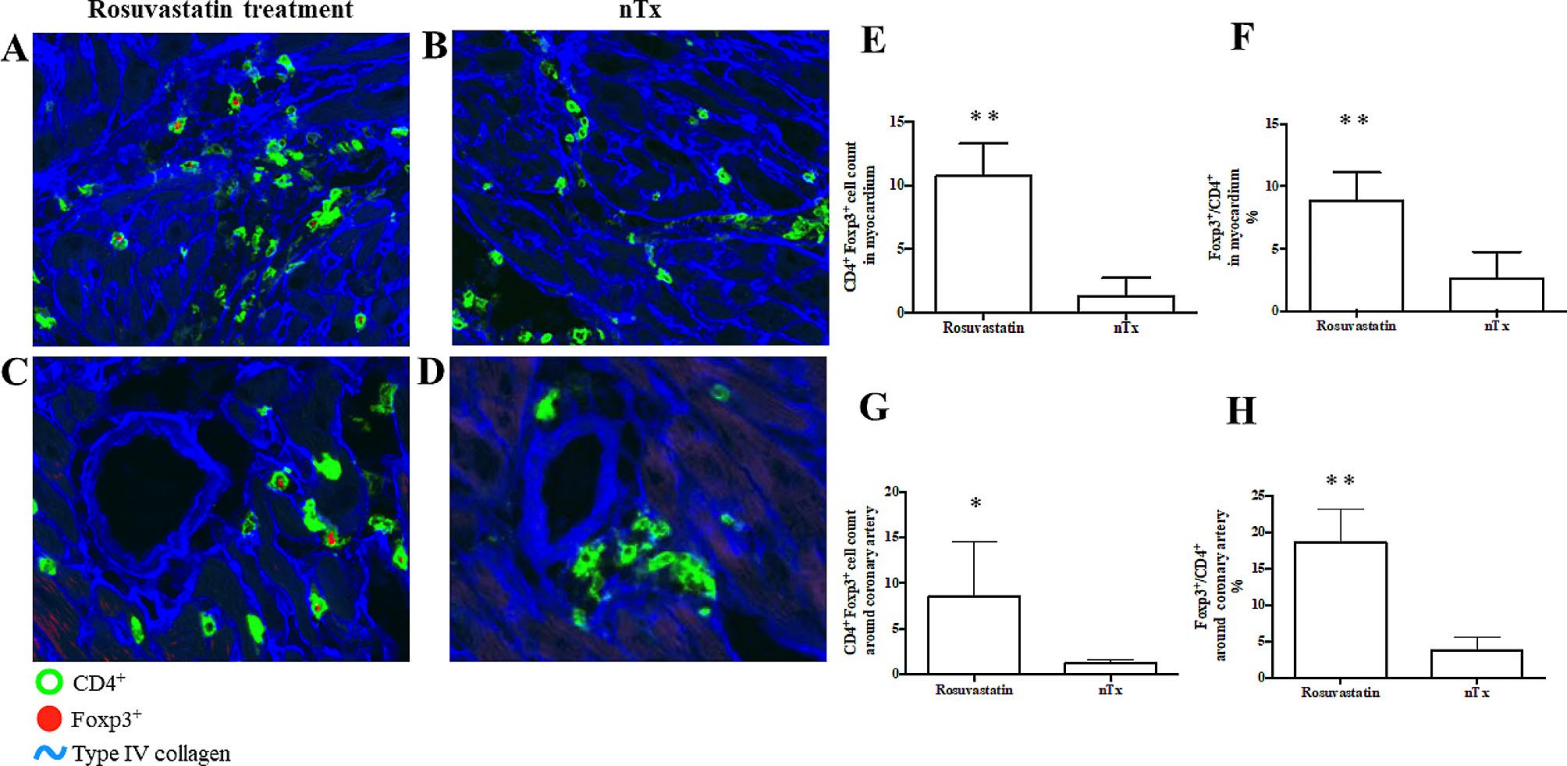
Triple fluorescent staining studies of cardiac allograft exposed with rosuvastatin. (**A**-**D**) Triple fluorescent staining studies of the myocardium (**A**, **B**) and coronary arteries (**C**, **D**) in cardiac allografts from rosuvastatin-treated and no-treatment recipients (nTx) were assessed two weeks after grafting. CD4, Foxp3, and type IV collagen are shown as green, red, and blue, respectively. (**E**, **F**) The number (**E**) and percentage (**F**) of infiltrating CD4^+^Foxp3^+^ cells in an area of 400 μm × 400 μm of the myocardium of cardiac allografts from each group. (**G**, **H**) The number (**G**) and percentage (**H**) of infiltrating CD4^+^Foxp3^+^ cells in an area of 75 μm × 75 μm of the around the coronary arteries of cardiac allografts from each group. The representative data are shown since we achieved similar results in these independent experiments. nTx, no-treatment. **P < 0.01 and*P < 0.05 compared with the no-treatment group

Fluorescent staining studies performed two weeks after grafting clearly demonstrated that cardiac allografts from rosuvastatin-treated CBA recipients showed aggregation of more CD4^+^Foxp3^+^ cells in the myocardium (Fig. 3A and B) and around the coronary arteries (Fig. 3C and D) of the allograft compared to those from no-treatment CBA recipients. The number of CD4^+^Foxp3^+^ cells and the percentage of Foxp3^+^ cells in CD4^+^ cells in myocardium and around the coronary arteries of the cardiac allografts from rosuvastatin-treated recipients obviously increased than those from no-treatment recipients (Fig. 3E-H).

### Generation of CD4^+^CD25^+^Foxp3^+^ Tregs in CBA recipients treated with rosuvastatin

**Fig. 4:**
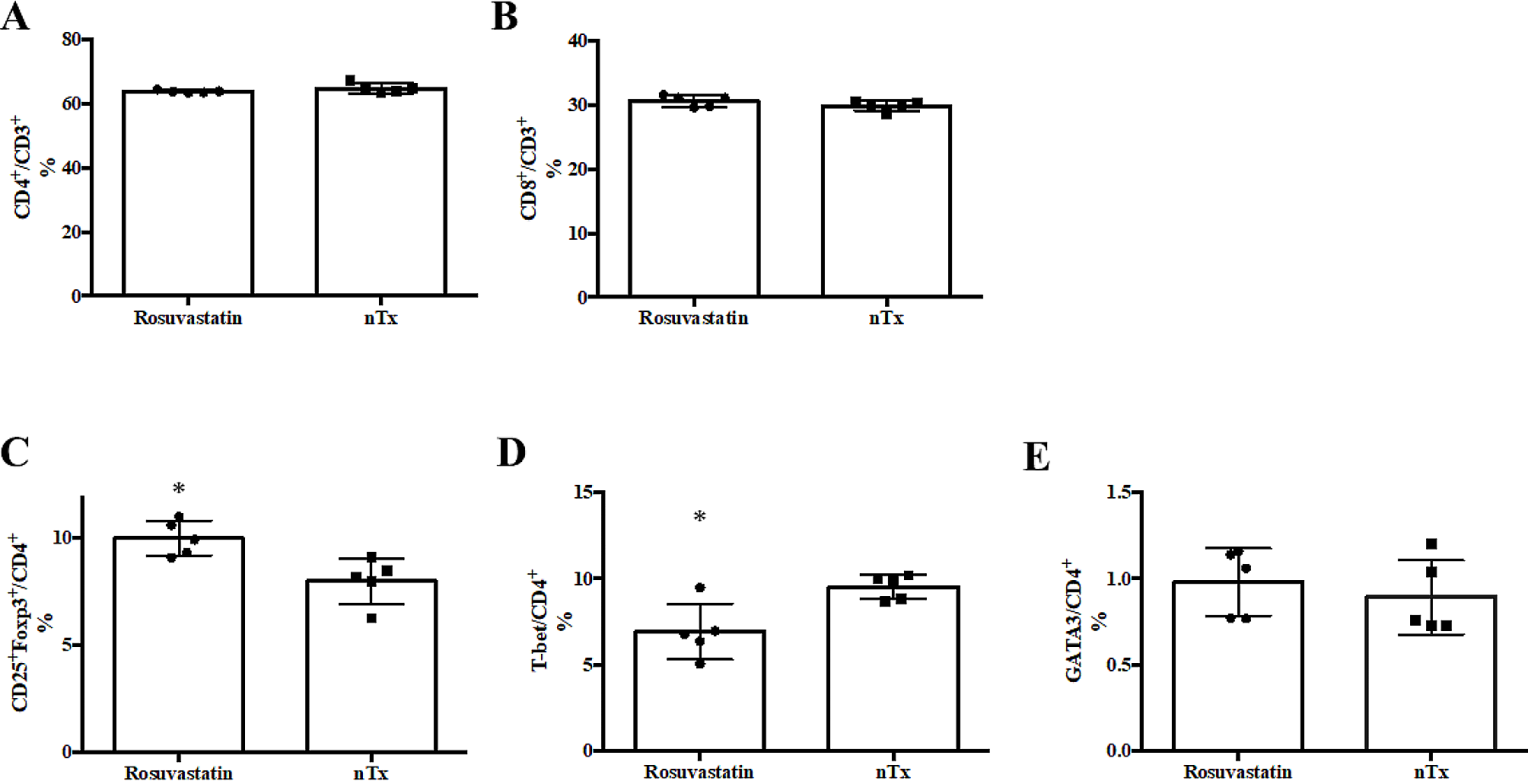
Flow cytometry analysis. The expression of CD4 (**A**), CD8 (**B**), CD4/CD25/Foxp3 (**C**), T-bet (**D**), and GATA3 (**E**) in splenocytes was determined by flow cytometry two weeks after transplantation. Data are represented as mean ± standard deviation of five animals in one experiment since we repeated flow cytometry experiment three times and achieved similar results in these independent experiments. nTx, no-treatment. *P < 0.05 compared with the no-treatment group

Flow cytometry studies showed that the population of splenic CD4^+^ or CD8^+^ T cells from rosuvastatin-treated CBA recipients was the same as that from no-treatment CBA recipients (Fig. 4A and B), whereas the population of splenic CD4^+^CD25^+^Foxp3^+^ T cells from rosuvastatin-treated CBA recipients increased compared to those of no-treatment CBA recipients (Fig. 4C).

### Change of intracellular T-bet/GATA3 expression of helper T (Th)-1 and Th-2 cells by rosuvastatin treatment

To assess the change in the Th-1 and Th-2 balance in rosuvastatin-treated CBA recipients with Tregs, intracellular T-bet and GATA3 specifically expressed in Th-1 and Th-2 cells were analyzed by flow cytometry. The number of CD4^+^T-bet^+^ T cells in splenocytes of rosuvastatin-treated CBA recipients was suppressed (Fig. 4D). However, there was no difference in CD4^+^GATA3^+^ T cells between the two groups (Fig. 4E), suggesting that rosuvastatin treatment inactivated Th-1 cells, which may render the Th-2 response more predominant.

### Cell proliferation by rosuvastatin and rosuvastatin-induced Treg

**Fig. 5:**
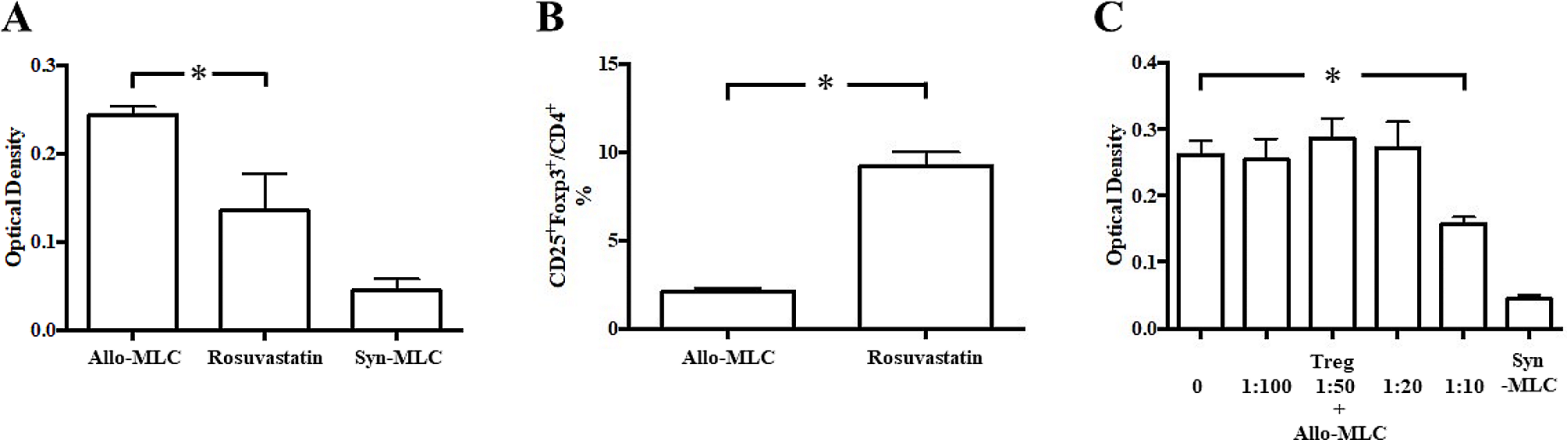
Cell proliferation analysis. (**A**) Direct effect of rosuvastatin on alloproliferation in the mixed leukocytes culture (MLC). The allogeneic MLC (allo-MLC) showed proliferation of CBA responder cells against B6 stimulator cells. The syngeneic MLC (syn-MLC) showed a minimal proliferation of responder cells alone (negative control). (**B**) The population of CD4^+^CD25^+^Foxp3^+^ T cells in the allogeneic MLC. (**C**) Various amounts of CD4^+^CD25^+^ splenocytes purified from rosuvastatin-treated recipients that had undergone transplantation of a B6 heart 14 days earlier were added to a fixed number of no-treatment CBA responders (2.5 × 10^5^/well) and B6 stimulators (5.0 × 10^5^/well). In MLC, we had five animals in each group and the MLC experiments were repeated three times. In each MLC, three replicates were made for each animal. Data are represented as mean ± standard deviation of five animals in one representative experiment since we repeated MLC experiment three times and achieved similar results in these independent experiments. MLC, mixed leukocyte culture. *P < 0.05 compared with the no-treatment group

The maximum proliferation of no-treatment CBA splenocytes (responder cells) against B6 splenocytes (stimulator cells) treated with mitomycin C was observed on the seventh day of MLC. The addition of rosuvastatin to allogeneic MLC inhibited the proliferation of CBA responder cells against B6 stimulator cells (Fig. 5A). In addition, the population of CD4^+^CD25^+^Foxp3^+^ T cells in the allogeneic MLC significantly increased in the rosuvastatin-added wells compared to that in the control wells (Fig. 5B). Moreover, the addition of rosuvastatin-induced CD4^+^CD25^+^ Tregs to allogeneic MLC showed no immunosuppressive effect at doses of 1:100 to 1:20 but had a significant cytosuppressive effect at doses of 1:10 (Fig. 5C).

### Attenuation of rosuvastatin treatment by PC-61

**Fig. 6:**
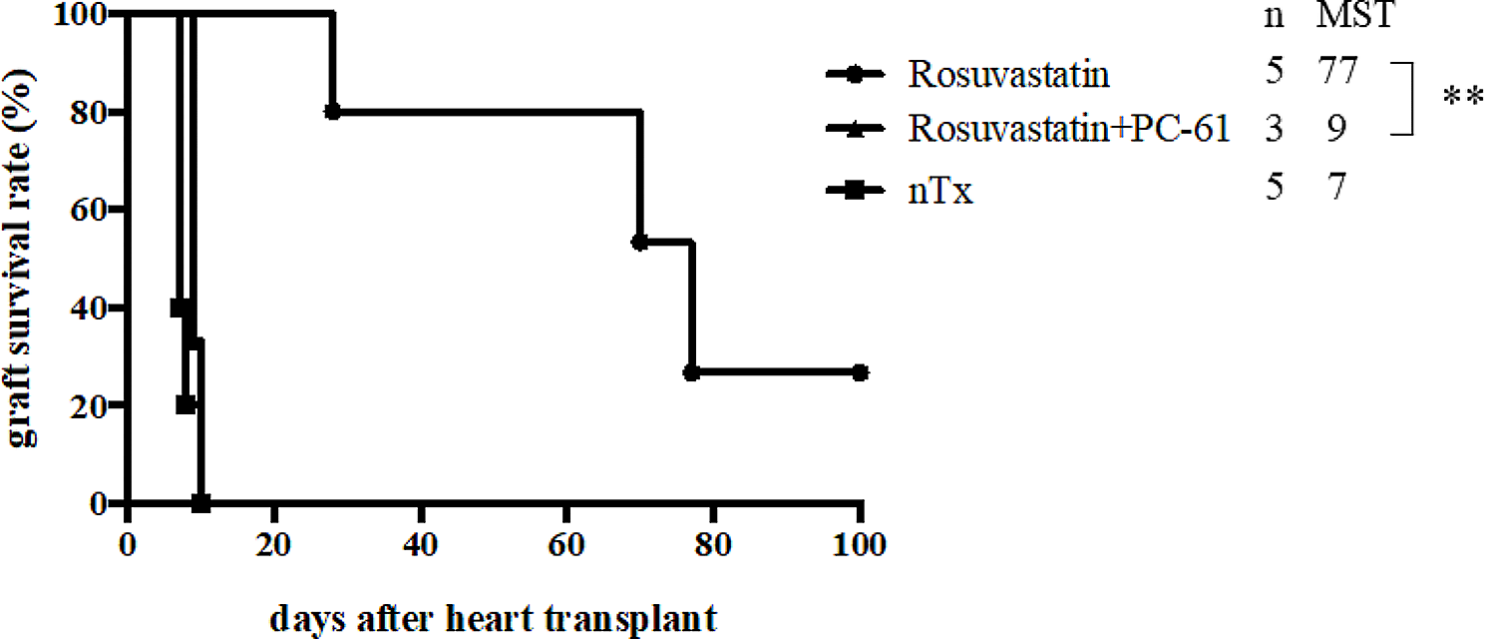
Administration of an anti-interleukin-2 receptor alpha monoclonal antibody (PC-61). Rosuvastatin-treated CBA recipients were administered 100 μg/d of PC-61 on days 0, 3, 6, and 9. MST, median survival time. PC-61, anti-interleukin-2 receptor alpha monoclonal antibody. **P < 0.01

Rosuvastatin-treated recipients with four doses of PC-61 displayed significantly shortened allograft survival compared with rosuvastatin-treated recipients (MST of 9 vs. 77 days; Fig. 6). It suggests that rosuvastatin-induced Treg may require the prolongation of graft survival.

### Suppression of DSA by rosuvastatin-induced Treg

**Fig. 7:**
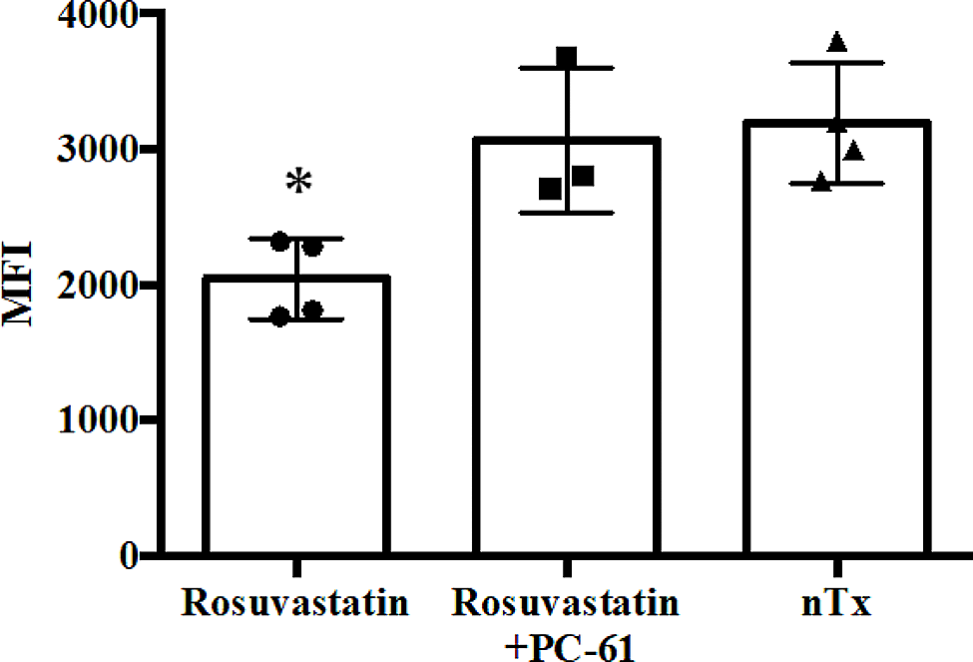
Assessment of donor-specific antibodies (DSAs). DSAs in the sera of rosuvastatin- and rosuvastatin/PC-61-treated CBA recipients were measured 14 days after transplantation. Data are shown as mean ± standard deviation of three to four animals in one experiment. MFI, mean fluorescence intensity. nTx, no-treatment. *P < 0.05 compared with the no-treatment group

DSA levels were downregulated on day 14 in CBA recipients treated with rosuvastatin compared to those in no-treatment CBA recipients (Fig. 7). Third-party (BALB/c) allospecific antibodies were not detected. Furthermore, CBA recipients treated with rosuvastatin and four doses of PC-61 (days 0, 3, 6, and 9) showed similarly upregulated DSA levels compared to no-treatment CBA recipients (Fig. 7). Taken together, these findings indicate that rosuvastatin treatment itself does not suppress DSA production but significantly promotes Treg populations that may drive sustained DSA suppression.

## Discussion

In this study, we present novel findings demonstrating the immunoprotective potential of rosuvastatin in the context of heart transplantation. The administration of rosuvastatin showed graft-protective effects. Furthermore, it significantly prolonged the survival of fully MHC-mismatched cardiac allografts and suppressed DSA production by upregulating CD4^+^CD25^+^Foxp3^+^ Tregs. Moreover, the addition of rosuvastatin to MLC suppressed cell proliferation by increasing the number of CD4^+^CD25^+^Foxp3^+^ Tregs. Our findings, along with those of previous reports, suggest several possible mechanisms by which rosuvastatin promotes cardiac allograft survival.

The main mechanism underlying the protective effects of cardiac allografts and the prolonged effects of rosuvastatin may involve the generation of Tregs. Among the many mechanisms of allograft hyporesponsiveness, immune regulation and the control of alloimmune responses by Tregs is thought to be one of the most important. Active suppression by Tregs, which drives mechanisms such as the modulation of dendritic cell function, inhibitory cytokine release, cytolysis, and metabolic disruption, is involved in the induction and maintenance of self-tolerance and unresponsiveness toward the allograft [25, 26]. Treg induction is a significantly important factor for better engraftment of cardiac allografts during the acute phase after grafting [25]. We have previously shown that herbal agents [27–29], treadmill exercise [22], thrombomodulin [30], and auditory stimulation [31] can induce alloreactive hyporesponsiveness in fully allogeneic grafts by generating Tregs. In this study, flow cytometry analysis indicated that the proportion of CD25^+^Foxp3^+^ Tregs within the CD4^+^ T cell population increased in transplant recipients treated with rosuvastatin relative to no-treatment recipients (Fig. 4C). The cardiac allografts from rosuvastatin-treated recipients contained more CD4^+^Foxp3^+^ cells than those from no-treatment recipients (Fig. 3E-H). Additionally, administration of PC-61 for CD25^+^ depletion to rosuvastatin-treated recipients impaired prolongation of cardiac allograft survival (Fig. 6). The addition of rosuvastatin to allogeneic MLC inhibited cell proliferation (Fig. 5A), which may have been driven by the promotion of Tregs (Fig. 5B). In the in vitro suppression test, the addition of rosuvastatin-induced CD4^+^CD25^+^ Tregs to allogeneic MLCs showed a significant cytosuppressive effect at a dose of 1:10. These findings were similar to the in vivo FACS results (Fig. 5C). Hence, consistent with previous reports demonstrating a relationship between Treg induction and statins [32, 33], our findings suggested that cardiac allograft survival may be promoted by the upregulation of Treg populations.

In addition to this possible mechanism of rosuvastatin-induced hyporesponsiveness and Treg induction, cholesterol regulation by rosuvastatin may have a strong influence on the prolongation of cardiac allografts. Treg stability and suppressive pathways are involved in intracellular mevalonate pathways involved in cholesterol synthesis [34]. In addition, one report demonstrated that atorvastatin-stimulated CD4^+^CD25^high^ and CD4^+^CD25^+^Foxp3^+^ T cells expressed high levels of Foxp3, which correlated with enhanced suppressive potential [35]. Although a direct comparison could not be performed, this was in line with our survival data collected after rosuvastatin and/or MVA administration. The results demonstrated that MVA administration mitigated the significant prolongation of cardiac allograft survival induced by rosuvastatin treatment (Fig. 1B and C), suggesting that cholesterol regulation via the mevalonate pathway was a significant factor affecting Treg stimulation and graft protection.

Another possible mechanism for rosuvastatin-induced hyporesponsiveness and Treg induction involves the strong influence of rosuvastatin-stimulated Tregs on the balance between Th-1 and Th-2 functions. The generation of Tregs correlates with a notable down-regulation of Th-1 function and upregulation of Th-2 function [36–38]. In a study on immune responses in atherosclerosis associated with cholesterol levels, statin therapy was shown to enhance Th-2 function and suppress Th-1 function [39]. In the present study, the number of splenic CD4^+^T-bet^+^ cells in rosuvastatin-treated recipients were significantly suppressed (Fig. 4D). Taken together, these findings indicate that treatment with rosuvastatin may inactivate Th-1 cells, which may make the Th-2 response more predominant and subsequently facilitate CD4^+^CD25^+^Foxp3^+^ Treg induction.

Another mechanism of graft-protective effects in rosuvastatin-treated recipients may involve the suppression of DSA production via rosuvastatin-induced Tregs rather than rosuvastatin itself. DSA is a key component in the diagnosis of acute and chronic AMR in organ transplants [40]. AMR is now considered to be progressively leading to the development of acute and chronic allograft damage, dysfunction, and loss [4]. Regarding the relationship between AMR and Treg, several recent studies have reported that donor-specific IgG-driven acute AMR in a murine kidney transplant model was reduced by Treg induction [41], and Foxp3^+^ Treg depletion in a murine lung allograft model has been reported to result in the generation of DSAs and AMR [42]. Additionally, the reduction in follicular Tregs in kidney transplant recipients was correlated with an increase in DSA production and chronic AMR disease progression [43]. In this study, DSA levels were downregulated on day 14 in CBA recipients treated with rosuvastatin compared to those in no-treatment CBA recipients (Fig. 7). Furthermore, consistent with previous reports demonstrating the effects of Treg-mediated DSA production, DSA increased to levels comparable to those in no-treatment recipients when PC-61 was administered on days 0, 3, 6, and 9 (Fig. 7). Collectively, although a more in-depth analysis to examine the correlation between prolonged survival and the suppression of DSA production is necessary, these findings suggest that the upregulation of Tregs by rosuvastatin is required to sustain the suppression of DSA in our model.

Finally, evidence suggests that rosuvastatin exerts direct protective effects on myocardial cell integrity and structure but not via Treg-mediated graft-protective effects. In a murine model, rosuvastatin administration proved to be effective against endothelial cell apoptosis [44] and showed broader effects on leukocyte function, such as modulation of cytokine and chemokine release [45]. In this study, histological analysis of cardiac allografts obtained 14 days after transplantation from rosuvastatin-treated recipients showed less leukocyte infiltration, maintenance of myocardial structure, and milder obliterative vasculopathy than those from no-treatment recipients (Fig. 2A and B). Taken together with these previous reports, our results indicate that rosuvastatin may exert graft-protective effects via the direct inhibition of alloreactive leukocytes, either through protection against endothelial cell apoptosis, anti-inflammatory activities, or other unknown mechanisms.

## Conclusion

Our findings demonstrate that rosuvastatin treatment may be a complementary graft-protective strategy to suppress DSA production in the acute phase, which may be driven by the promotion of splenic and graft-infiltrating Tregs and other immunomodulatory mechanisms.

## Data Availability

The datasets used are available from the corresponding author on reasonable request.
